# Clinical Interest of Combining Transcriptomic and Genomic Signatures in High-Grade Serous Ovarian Cancer

**DOI:** 10.3389/fgene.2020.00219

**Published:** 2020-03-17

**Authors:** Yann Kieffer, Claire Bonneau, Tatiana Popova, Roman Rouzier, Marc-Henri Stern, Fatima Mechta-Grigoriou

**Affiliations:** ^1^Institut Curie, Stress and Cancer Laboratory, Equipe labelisée Ligue Nationale Contre le Cancer, PSL University, Paris, France; ^2^Inserm, U830, Paris, France; ^3^Genomics and Biology of Hereditary Cancers, Institut Curie, Paris, France; ^4^Department of Surgery, Institut Curie Hospital Group, René Huguenin Hospital, Saint-Cloud, France

**Keywords:** HGSOC, fibrosis, mesenchymal, BRCA1/2, homologous recombination deficiency, prognosis

## Abstract

High-grade serous ovarian cancer is one of the deadliest gynecological malignancies and remains a clinical challenge. There is a critical need to effectively define patient stratification in a clinical setting. In this study, we address this question and determine the optimal number of molecular subgroups for ovarian cancer patients. By studying several independent patient cohorts, we observed that classifying high-grade serous ovarian tumors into four molecular subgroups using a transcriptomic-based approach did not reproducibly predict patient survival. In contrast, classifying these tumors into only two molecular subgroups, fibrosis and non-fibrosis, could reliably inform on patient survival. In addition, we found complementarity between transcriptomic data and the genomic signature for homologous recombination deficiency (HRD) that helped in defining prognosis of ovarian cancer patients. We also established that the transcriptomic and genomic signatures underlined independent biological processes and defined four different risk populations. Thus, combining genomic and transcriptomic information appears as the most appropriate stratification method to reliably subgroup high-grade serous ovarian cancer patients. This method can easily be transferred into the clinical setting.

## Introduction

Epithelial ovarian cancer is the fifth leading cause of cancer-related death among women, with only 40% of patients achieving an average 5-year survival (Berns and Bowtell, 2012). Ovarian cancers are predominantly classified by histological subtype (serous, endometrioid, mucinous, clear cell or squamous), grade (low or high) and stage (early or advanced). Approximately 75% of ovarian cancers are high-grade serous ovarian cancers. Standard treatment consists of surgical cytoreduction combined with Taxanes- and platinum salts-based chemotherapy. Recently, targeted therapies have also been included in treatment plans, such as anti-angiogenic drugs or poly-ADP-ribose polymerase (PARP) inhibitors indicated for certain patients with BRCA1/2 mutations (Fong et al., 2009, 2010; Tutt et al., 2010; Gelmon et al., 2011; Kaye et al., 2012; Ledermann et al., 2012; Liu et al., 2014; Oza et al., 2015; Konecny and Kristeleit, 2016; McLachlan et al., 2016; Miller and Ledermann, 2016). Novel targeted therapies are being developed but their use remains limited, in part due to their cost (Raja et al., 2012; Kmietowicz, 2015; Monk et al., 2016; The Lancet, 2017). To increase the effectiveness of targeted therapies, there is a need to develop accurate methods to define novel patient stratifications that can be easily translated to the clinical environment.

Ovarian cancers have a high frequency of homologous recombination deficiency (HRD) due to germline or somatic mutations in the BRCA1 or BRCA2 genes, methylation of the BRCA1 or RAD51C promoter regions or other genetic alterations (Rigakos and Razis, 2012; Muggia and Safra, 2014). Patients carrying BRCA1/2 mutations have increased sensitivity to platinum salts and longer survival than patients with no BRCA1/2 mutations (Fong et al., 2009, 2010; Audeh et al., 2010; Goundiam et al., 2015) and HRD sensitizes cells to PARP inhibitors (Pujade-Lauraine et al., 2017). To assess HRD in breast and ovarian cancer, the large-scale state transition (LST) genomic signature can be used (Popova et al., 2012; Goundiam et al., 2015). In addition to genomic characterization, previous studies have identified distinct molecular subgroups of high-grade serous ovarian cancers based on transcriptomic profiling (Tothill et al., 2008; Cancer Genome and Atlas Research, 2011; Mateescu et al., 2011; Sabatier et al., 2011; Bentink et al., 2012; Verhaak et al., 2013; Konecny et al., 2014). Importantly, all currently published studies observed one molecular subgroup, referred to as Stromal, Fibrotic, Mesenchymal or Angiogenic, that is invariably associated with poor patient survival (Tothill et al., 2008; Mateescu et al., 2011; Bentink et al., 2012; Verhaak et al., 2013; Konecny et al., 2014). The first mechanism that explains the Fibrotic/Mesenchymal subgroup, at least in part, is regulation by the miR-200 family of microRNAs (Mateescu et al., 2011; Batista et al., 2013, 2016). Genes inversely correlated with expression of the miR-200 family constitute the fibrosis signature that classifies ovarian cancers with mesenchymal features (Mateescu et al., 2011; Batista et al., 2013, 2016). Conversely, genes positively-correlated with miR-200 expression constitute an oxidative stress signature that classifies the oxidative stress ovarian cancer subgroup. This stress subgroup is associated with a better prognosis and increased cancer cell chemosensitivity (Leskela et al., 2011; Mateescu et al., 2011; Batista et al., 2013, 2016; Brozovic et al., 2015). Notably, the accumulation of miR-200 family members in ovarian tumors could be used for early detection of the pathology, but determining patient outcome through miR-200 expression remains highly controversial, and a consensus is far from being achieved (Batista et al., 2013; Muralidhar and Barbolina, 2015; Shi and Zhang, 2016). The ability to provide information on patient survival remains a priority in the field but the number of molecular subgroups required to define patient survival effectively is unknown, impeding their use in clinical practice. In this study, we address this question and define the optimal number of ovarian cancer molecular subgroups for prognostic stratification of patients.

## Materials and Methods

### Clinical and Transcriptomic Data of Ovarian Cancer Patients

Three cohorts of patients with high-grade serous ovarian cancer were included in this study: Curie, AOCS and TCGA. Curie cohort: Ovarian tumors were obtained from a cohort of 107 patients treated at the Institut Curie between 1989 and 2005. Clinical characteristics of the cohort have already been described in Mateescu et al. (2011). For each patient, a surgical specimen was taken, prior to any chemotherapeutic treatment, for pathological analysis and tumor tissue cryopreservation. The median patient age was 58 years old (with a range of 31–87 years). Ovarian carcinomas were classified according to the World Health Organization histological classification of gynecological tumors. The Curie transcriptomic dataset is from Affymetrix Human Genome U133 Plus 2.0 arrays and is freely available in the Gene Expression Omnibus^1^ under the accession number, GSE26193. AOCS cohort: Clinical characteristics of the 285 patients included in the AOCS cohort have been previously described in Tothill et al. (2008), and transcriptomic data, generated using Affymetrix Human Genome U133 Plus2.0 arrays, are freely available under the accession number, GSE9899. TCGA cohort: Clinical characteristics of the 557 patients included in the TCGA cohort, as well as transcriptomic data generated using Affymetrix Human Genome U133A arrays, have been previously described in Cancer Genome and Atlas Research (2011) and can be downloaded from the NIH Genomic Data Commons (GDC) data portal^2^. Most patients treated at Institut Curie are from Caucasian origin and 91% of the patients, for which the ethnicity variable is known in the TCGA cohort, are also from Caucasian origin.

### Description of Transcriptomic Signatures

Transcriptomic signatures defining the molecular classification of ovarian cancers were retrieved from four original publications. First, Tothill et al. (2008) identified 478 Affymetrix HG U133 Plus 2.0 probe sets up-regulated in the C1 signature and 2,230 probe sets up-regulated in the C2–C6 signature. Second, Mateescu et al. (2011) identified 22 genes up-regulated in the Stress/non-Fibrosis signature and 16 genes up-regulated in the Fibrosis signature. Third, Bentink et al. (2012) identified 100 Illumina probes up-regulated in the M1 signature and 300 Illumina probes up-regulated in the M2–M4 signature. Lastly, Verhaak et al. (2013) identified 37 genes up-regulated in the Mesenchymal signature and 63 genes up-regulated in the Differentiated/Immunoreactive/Proliferative signature. The different transcriptomic signatures coming from these distinct studies are not overlapping in terms of genes (as shown Supplementary Figures S1B,D), enabling us to compare these different signatures as distinct entities.

### Enrichment of Biological Processes in Transcriptomic Signatures

Gene ontology (GO) enrichment analysis was performed using the DAVID bioinformatics resources (Version 6.7)^3^. For each signature tested, the 10 most significant biological processes (based on *p*-value) were selected. Reduce and Visualize Gene Ontology (REViGO) software (Supek et al., 2011; accessed January 2017)^4^, with a parameter similarity of 0.5, was used to summarize information by removing redundant GO terms.

### Classification of High-Grade Serous Ovarian Cancer From the TCGA Cohort According to Different Transcriptomic Signatures

**FIGURE 1:**
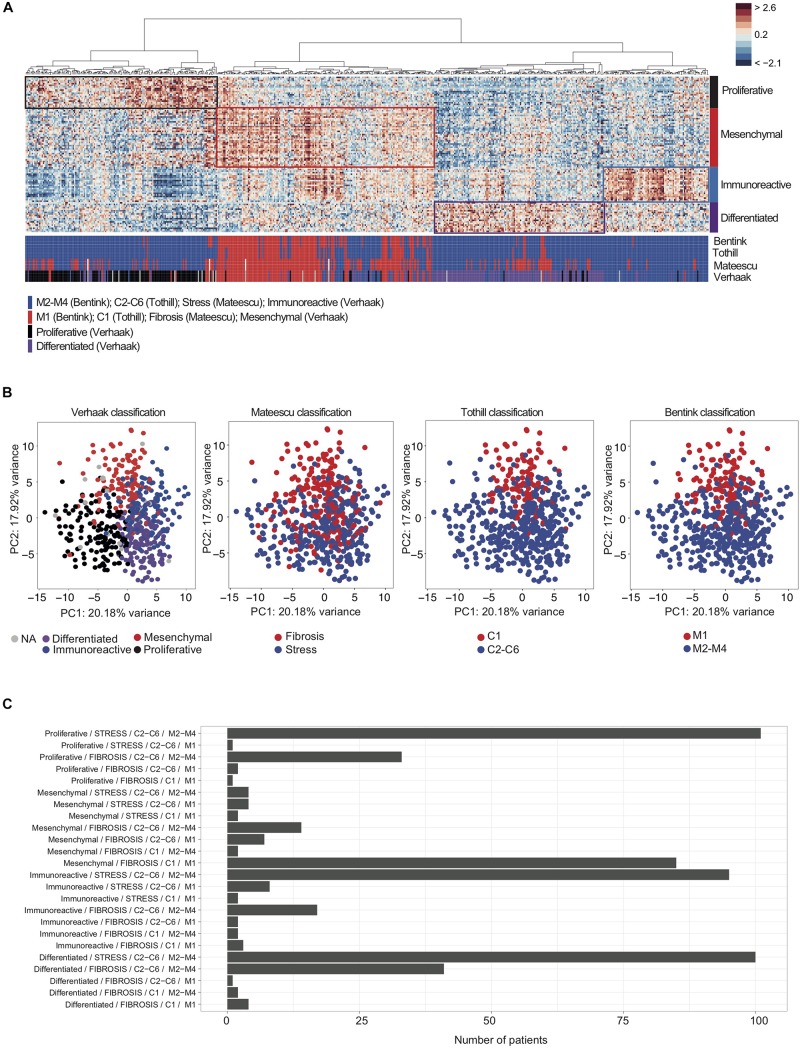
Overlap between transcriptomic signatures used for classification of high-grade serous ovarian cancers. **(A)** A heatmap from hierarchical clustering applied on the TCGA cohort. Rows represent genes and columns represent patients. Clustering is based on the 100 genes of the D-I-M-P signature (Verhaak et al., 2013) using Pearson distance and Ward’s agglomeration method. The color saturation shows the magnitude of the deviation from the mean for each gene, with red and blue indicating expression values above or below the mean, respectively. Colored bars below the heatmap represent tumor classifications obtained from the four (transcriptomic signatures (Tothill et al., 2008; Mateescu et al., 2011; Bentink et al., 2012; Verhaak et al., 2013), as indicated. The red bars correspond to the Mesenchymal, C1, Angiogenic or Fibrosis subgroup, according to the classification considered. The blue bars correspond to C2–C6, non-Angiogenic and Stress subgroups. For the D-I-M-P signature, blue bars correspond to Immunoreactive, black correspond to Proliferative and purple bars correspond to Differentiated subgroups. **(B**, Left) Principal Component Analysis (PCA) applied on transcriptomic data from the TCGA cohort, using the 100 genes composing the D-I-M-P signature (Verhaak et al., 2013). The color code represents the four D-I-M-P molecular subgroups: Mesenchymal (red, *N* = 118), Differentiated (purple, *N* = 148), Proliferative (black, *N* = 138) and Immunoreactive (blue, *N* = 129). (Middle and Right) Further PCA with subgroups highlighted using Fibrosis (red, *N* = 220) or Stress (blue, *N* = 326) (Mateescu et al., 2011); C1 (red, *N* = 107) or C2–C6 (blue, *N* = 443) (Tothill et al., 2008); Angiogenic (M1, red, *N* = 128) or non-Angiogenic (M2–M4, blue, *N* = 422) (Bentink et al., 2012) signatures, as indicated. **(C)** Barplots showing the number of patients according to each combination of classes among the four classifications (Verhaak/Mateescu/Tothill/Bentink).)

High-grade serous ovarian cancers from the TCGA (Cancer Genome and Atlas Research, 2011) were studied (see Table 1 for cohort description). Genes that comprise the C1–C6 (Tothill et al., 2008), Stress/Fibrosis (Mateescu et al., 2011), and M1–M4 (Bentink et al., 2012) signatures were applied to the TCGA transcriptomic data. This allowed us to classify high-grade serous ovarian cancers from the TCGA cohort according to Tothill’s, Mateescu’s, and Bentink’s signatures, and compare them to the Differentiated/Immunoreactive/Mesenchymal/Proliferative (D-I-M-P) classification, initially generated from the TCGA dataset (Verhaak et al., 2013). Briefly, we first performed the hierarchical clustering shown in Figure 1A based on DIMP signature using Euclidean distance and Ward’s agglomeration method. To compare this DIMP classification with the others, we next performed similar hierarchical clustering by applying each of the other signatures (Tothill et al., 2008; Mateescu et al., 2011; Bentink et al., 2012) on the TCGA transcriptomic dataset by using same parameters (Euclidean distance and Ward’s agglomeration method). Only genes specific of each signatures were kept for the clustering. For the four signatures, each resulting dendrogram tree was next cut into two subgroups for classifying patients into two subgroups according to each signature (Stress/Fibrosis for Mateescu classification, C1/C2–C6 for Tothill classification and M1/M2–M4 for Bentink classification). By this way, for each of the four classifications studied, we have been able to determine to which subgroup each patient belongs, as show Figures 1A,B. The distribution of ovarian cancers from TCGA across the four signatures can be found in Table 1. Patient classification was thus independent of patient survival and strictly based on tumor molecular signature. We also aimed at comparing the association of patient clinical features with two distinct classifications, i.e., classification in two subgroups based on Mateescu’s signature and classification in four subgroups based on Verhaac’s signature using Fisher’s exact test (as shown in Table 2). No correction was applied to *p*-values.

**TABLE 1:** Comparative description of clinical parameters in the AOCS, Curie, and TCGA cohorts.

		**AOCS**	**Curie**	**TCGA**
Total number of patients		285	107	557
Age (year)	Median	59	58	59
	Range	22–80	31–87	26–89
Histotype				
	Serous	264 (92.6%)	82 (76.6%)	557 (100%)
	Endometrioid	20 (7.02%)	8 (7.5%)	
	Adenocarcinoma	1 (0.4%)		
	Mucinous		8 (7.5%)	
	Other		9 (8.4%)	
Figo Substage				
	I	24 (8.4%)	21 (19.6%)	
	II	18 (6.3%)	10 (9.35%)	24 (4.3%)
	III	217 (76.1%)	59 (55.14%)	381 (68.4%)
	IV	22 (7.7%)	17 (15.9%)	79 (14.2%)
	Not applicable	4 (1.4%)		73 (13.1%)
Grade				
	1	19 (6.7%)	7 (6.5%)	
	2	97 (34%)	34 (31.5%)	57 (10.2%)
	3	164 (57.5%)	66 (62%)	420 (75.4%)
	Not applicable	5 (1.8%)		80 (14.4%)
Surgery				
	Full	84 (29.5%)	38 (35.5%)	90 (16.2%)
	Partial	164 (57.5%)	69 (64.5%)	342 (61.4%)
	Not applicable	37 (13%)		125 (22.4%)
Clinical response	RC – complete response		51 (47.7%)	276 (49.6%)
	RP – Partial response		22 (20.6%)	57 (10.2%)
	S – Stability		7 (6.5%)	25 (4.5%)
	P – Progression		11 (10.3%)	37 (6.6%)
	Not applicable	285 (100%)	16 (15%)	162 (29.1%)
Signature D-I-M-P				
	Differentiated		30 (28%)	148 (26.6%)
	Immunoreactive		26 (24.3%)	129 (23.2%)
	Mesenchymal		31 (29%)	118 (21.2%)
	Proliferative		20 (18.7%)	138 (24.8%)
	Not applicable	285 (100%)		24 (4.3%)
Mateescu’s Signature				
	Stress	150 (52.6%)	51 (47.7%)	326 (58.5%)
	Fibrosis	135 (47.4%)	56 (52.3%)	220 (39.5%)
	Not applicable			11 (2%)
Tothill’s Signature				
	C1	83 (29.1%)		107 (19.2%)
	C2–C6	168 (58.9%)		443 (79.5%)
	Not applicable	34 (11.9%)		7 (1.3%)
Bentink’s Signature				
	M1			128 (23%)
	M2–M4			422 (75.8%)
	Not applicable	285 (100%)		7 (1.3%)
Lst Signature				
	Low LST			238 (42.7%)
	High LST			303 (54.4%)
	Not applicable	285 (100%)		16 (2.9%)

**AOCS, Curie, and TCGA cohorts have previously been described in Tothill et al. (2008), Cancer Genome and Atlas Research (2011), and Mateescu et al. (2011), respectively. For the Curie cohort, tumor samples were obtained from a cohort of ovarian carcinoma patients treated at the Institut Curie from 1989 to 2012. For each patient, a surgical specimen was taken, prior to any chemotherapeutic treatment, for pathological analysis and tumor tissue cryopreservation. The median patient age was 58 years old (with a range of 31–87 years). Ovarian carcinomas were classified according to the World Health Organization histological classification of gynecological tumors.**

**TABLE 2:** The association between transcriptomic signatures and clinical parameters.

		**Fibrosis/non-Fibrosis classification**				

		**Non-fibrosis**		**Fibrosis**		***p*-value**
Grade						*p* = 0.67
	G2	32 (11.6%)		25 (12.8%)		
	G3	245 (88.5%)		170 (87.2%)		
Stage						***p* = 0.01**
	II	20 (7.1%)		4 (2%)		
	III–IV	260 (92.9%)		195 (98%)		
Debulking						***p* = 0.05**
	Full	60 (24%)		28 (15.8%)		
	Partial	190 (76%)		149 (84.2%)		
Platinum resistance						*p* = 0.38
	Sensitive	153 (75.7%)		99 (71.3%)		
	Resistant	49 (24.3%)		40 (28.8%)		
Primary therapy outcome						***p* = 0.02**
	Complete response	172 (74.5%)		101 (63.1%)		
	Partial response	59 (25.6%)		59 (37%)		
BRCA1/2 mutation						*p* = 0.11
	No	269 (84.6%)		171 (78.8%)		
	Yes	49 (15.4%)		46 (21.2%)		
BRCA1 methylation						*p* = 1
	No	278 (87.4%)		190 (87.6%)		
	Yes	40 (12.6%)		27 (12.4%)		
RAD51C methylation						*p* = 0.39
	No	312 (98.1%)		210 (96.8%)		
	Yes	6 (1.9%)		7 (3.2%)		
LST signature (HRD)						*p* = 0.29
	Low	147 (46.2%)		89 (41.2%)		
	High	171 (53.8%)		127 (58.8%)		
Ploidy						*p* = 0.20
	2	104 (32.7%)		83 (38.4%)		
	≥ 4	214(67.3%)		133 (61.6%)		

			**D-I-M-P classification**				

		**D**	**I**	**M**	**P**	***p*-value**

Grade						*p* = 0.28
	G2	13 (10.1%)	9 (8.8%)	17 (17%)	18 (13.4%)	
	G3	116 (89.9%)	93 (91.2%)	83 (83%)	116 (86.6%)	
Stage						***p* = 0.007**
	II	5 (3.7%)	12 (11.5%)	1 (1%)	6 (4.5%)	
	III–IV	129 (96.3%)	92 (88.5%)	101 (99%)	126 (95.5%)	
Debulking						***p* = 0.03**
	Full	33 (26.8%)	17 (19.5%)	10 (11%)	28 (23.5%)	
	Partial	90 (73.2%)	70 (80.5%)	81 (89%)	91 (76.5%)	
Platinum resistance						*p* = 0.70
	Sensitive	70 (70%)	54 (78.3%)	53 (74.7%)	73 (74.5%)	
	Resistant	30 (30%)	15 (21.7%)	18 (25.4%)	25 (25.5%)	
Primary therapy outcome						*p* = 0.15
	Complete response	78 (69.6%)	60 (69%)	49 (62%)	85 (77.3%)	
	Partial response	34 (30.3%)	27 (31%)	30 (38%)	25 (22.7%)	
BRCA1/2 mutation						***p*** = 0.05
	No	118 (79.7%)	103 (79.8%)	94 (79.7%)	124 (89.9%)	
	Yes	30 (20.3%)	26 (20.2%)	24 (20.3%)	14 (10.1%)	
BRCA1 methylation						*p* = 0.15
	No	127 (85.8%)	110 (85.3%)	101 (85.6%)	128 (92.8%)	
	Yes	21 (14.2%)	19 (14.7%)	17 (14.4%)	10 (7.2%)	
RAD51C methylation						*p* = 0.38
	No	144 (97.3%)	124 (96.1%)	115 (97.5%)	137 (99.3%)	
	Yes	4 (2.7%)	5 (3.9%)	3 (2.5%)	1 (0.7%)	
LST signature (HRD)						***p*** = 0.0002
	Low	62 (41.9%)	43 (33.3%)	48 (40.7%)	82 (59.4%)	
	High	86 (58.1%)	86 (66.7%)	70 (59.3%)	56 (40.6%)	
Ploidy						***p*** = 4.6e-5
	2	71 (48.0%)	39 (30.2%)	46 (39.0%)	31 (22.5%)	
	≥ 4	77 (52.0%)	90 (69.8%)	72 (61.0%)	107 (77.5%)	

**Contingency table showing the association between Fibrosis/non-Fibrosis (two subgroups, Mateescu’s classification), or D-I-M-P subgroups (four subgroups, Verhaac’s classification), and clinical parameters. Data are from the TCGA cohort and the number of patients and frequencies in the population are indicated. Debulking status was defined as full when no macroscopic residue was detected after surgery or as partial otherwise. Response to primary therapy was considered as partial if the patient indicated with partial response, stable disease or progressive disease, considering both surgery efficiency and sensitivity to chemotherapies. P-values are calculated using Fisher’s exact test without correction and significant p-values are indicated in bold.**

### Expression of miR-200 Family Members

The predictive value of the miR-200 family was evaluated because this miRNA family was shown to be associated with the stress (non-Fibrosis)/Fibrosis classification (Mateescu et al., 2011; Batista et al., 2013, 2016). Indeed, genes that are inversely correlated with the miR-200 expression compose the “Fibrosis” signature and classify ovarian cancers with mesenchymal features. Conversely, genes positively-correlated with miR-200 expression constitute the non-Fibrosis (oxidative stress) signature and classify the “non-Fibrosis” ovarian cancer subgroup. Expression of the miR-200 family members (miR-141, miR-200a, miR-200b, miR-200c, and miR-429) was determined using the level 3 expression data from the TCGA data portal. Groups of low or high microRNA expression were defined using their median as a threshold to perform survival analysis.

### Large-Scale State Transition (LST) Genomic Signature of HRD

Cytoscan HD SNP-array (Affymetrix) data were processed using the Genome Alteration Print (GAP) methodology to obtain absolute copy number profiles (Popova et al., 2009). DNA index was calculated as the averaged copy number. Based on the DNA index, tumor ploidy was set as near-diploid (DNA index < 1.3) or near-tetraploid (DNA index ≥ 1.3). Detection of HRD was determined by the number of LST, as previously described (Popova et al., 2012). Briefly, LST was defined as a chromosomal breakpoint (change in copy number or major allele counts) between adjacent regions of at least 10 Mb. The number of LST were then calculated after smoothing and filtering out copy number variant regions < 3 Mb. Tumors were segregated into near-diploid or near-tetraploid subgroups. Based on two ploidy-specific cut-offs (15 and 20 LST per genome in near-diploid and near-tetraploid tumors, respectively) tumors were classified as LST high (LST^Hi^, equal or above the cut-off) or LST low (LST^Lo^, below the cut-off). LST^Hi^ represents the HRD genomic pattern and LST^Lo^ corresponds to the non-HRD profile.

### Statistical Analysis

All statistical analyses were performed in the R environment (Versions 3.3.2, 3.4.0, and 3.6.1)^5^. Fisher’s exact test was used to determine any association between classes of ovarian cancers and clinical parameters. Overall survival (OS) and disease-free survival (DFS) were investigated using the Cox proportional hazards model and Kaplan-Meier curves through the R packages, *survival* and *survminer*. To identify differences between survival curves, *p*-values were assessed by the log-rank test. *P*-values ≤ 0.05 were considered to be statistically significant. To take into account multiple testing, *p*-values were adjusted using the Benjamini-Hochberg procedure using *pairwise*_survdiff function from R package *Survminer*.

### Code Availability

R scripts used to generate panels of the Figures, Supplementary Figures and Tables are provided within the data source file of the paper, available with the DOI number: 10.6084/m9.figshare.11663232.

## Results

### The Fibrosis Subgroup of High-Grade Serous Ovarian Cancers Exhibits Conserved Functional Pathways Across Studies

Although the genes defining ovarian cancer molecular subgroups were different across studies (Tothill et al., 2008; Cancer Genome and Atlas Research, 2011; Mateescu et al., 2011; Sabatier et al., 2011; Bentink et al., 2012; Verhaak et al., 2013; Konecny et al., 2014), we observed that some of the identified functions were consistent across the fibrosis subgroups (Supplementary Figures S1A,B). Following a GO enrichment analysis on previously published ovarian cancer transcriptomic signatures (Tothill et al., 2008; Cancer Genome and Atlas Research, 2011; Mateescu et al., 2011; Bentink et al., 2012; Verhaak et al., 2013), we found consistent enrichment in particular pathways, including cell adhesion, extracellular matrix organization, and response to wounding (Supplementary Figure S1A). It is important to note that this molecular ovarian cancer subgroup was named differently across studies, and referred to as C1 (Tothill et al., 2008), Fibrosis (Mateescu et al., 2011), Angiogenic (Bentink et al., 2012), or Mesenchymal (Verhaak et al., 2013; Konecny et al., 2014) subgroups, but they all possess similar biological features (mainly fibrosis and mesenchymal properties) (Supplementary Figure S1A). However, apart from Fibronectin 1 (FN1), the transcriptomic signatures did not show any overlap in gene expression (Supplementary Figure S1B). In contrast to the C1/Fibrosis/Angiogenic/Mesenchymal signature, none of the others signatures, defining C2–C6 (Tothill et al., 2008), Oxidative stress (Mateescu et al., 2011), Anti-angiogenic (M2–M4) (Bentink et al., 2012), or Differentiated-Immunoreactive-Proliferative (Verhaak et al., 2013; Konecny et al., 2014) high-grade serous ovarian cancer subgroups, showed overlap in either gene expression or pathways (Supplementary Figures S1C,D).

We next sought to test if the ovarian cancer patients identified by these different transcriptomic signatures were the same (Figure 1). To do so, we studied the TCGA cohort (Cancer Genome and Atlas Research, 2011; Table 1 for cohort description) and classified each patient using the four transcriptomic signatures (Tothill et al., 2008; Mateescu et al., 2011; Bentink et al., 2012; Verhaak et al., 2013). Unsupervised analyses, including hierarchical clustering (Figure 1A) and Principal Component Analyses (Figure 1B), confirmed that there was a significant overlap between tumor classification in C1, Fibrosis, Angiogenic, and Mesenchymal subtypes. Indeed, patients classified as Mesenchymal were also mainly classified as Fibrosis, C1 and M1, while patients classified as Stress, C2–C6 and M1–M4 can be equally classified as Proliferative, Immunoreactive or Differentiated (Figure 1C). Therefore, these different gene signatures not only identified the same biological characteristics (mesenchymal properties, accumulation of extra-cellular matrix components, and pro-angiogenic features) but also identified the same patients (Figures 1A–C). Almost all patients (91.5%) defined as Mesenchymal (using Verhaak’s signature) were also classified as Fibrosis (using Mateescu’s signature). However, 26% of non-Mesenchymal patients (using Verhaak’s signature) were identified as Fibrosis (using Mateescu’s signature), suggesting possible misclassifications. Interestingly, the overall survival and disease-free survival of those discordant patients (non-Mesenchymal/Fibrosis) were similar to the Mesenchymal/Fibrosis defined patients, but significantly different from non-Mesenchymal/non-Fibrosis patients (Supplementary Figure S2). This suggests that these patients could be classified as Fibrosis, as defined by Mateescu’s signature. These observations show that high-grade serous ovarian cancers can be divided into two major molecular subtypes according to transcriptomic profiles: Fibrosis and non-Fibrosis.

### High-Grade Serous Ovarian Cancers Stratified Into Two Subgroups Are Associated With Stage, Debulking, and Clinical Response to Treatment

We next questioned if stratification of ovarian cancers into four molecular subgroups (such as D-I-M-P, based on Verhaak’s classification) could be more informative regarding clinical features than classification into two subgroups (Fibrosis and non-Fibrosis) (based on Mateescu’s classification). The non-Fibrosis and Fibrosis subgroups were significantly associated with stages (*p* = 0.01), debulking (*p* = 0.05) and primary therapy outcome (*p* = 0.02) (Table 2). However, they were not associated with grade, ploidy, sensitivity to platinum, BRCA1/2 mutations or BRCA1 or RAD51C promoter methylation (Table 2). LST signature, which is linked to HRD status (Fong et al., 2009, 2010; Audeh et al., 2010; Popova et al., 2012; Goundiam et al., 2015), was also not significantly associated with the Fibrosis and non-Fibrosis subgroups (Table 2). The four D-I-M-P subgroups showed a significant association with stage (*p* = 0.007) and debulking (*p* = 0.03), but not with response to treatment. In addition, D-I-M-P was associated with ploidy (*p* = 4.6e-5), BRCA1/2 mutations (*p* = 0.05) and LST signature (*p* = 0.0002) but not with grade, platinum resistance and primary therapy outcome (Table 2). These results suggest that defining Mesenchymal ovarian cancers by applying the D-I-M-P signature is less informative than the Fibrosis classification.

### Stratification Into Two Ovarian Cancer Subgroups Provides a Reliable Prognostic Value for Patient Survival

Taking into account the association between transcriptomic signatures and clinical parameters, we next investigated if these different transcriptomic signatures could be utilized as independent prognostic factors, compared to stage and debulking status, the two major variables of patient outcome used in clinics. Based on univariate analyses using the Cox regression model, we observed that the Fibrosis/non-Fibrosis signature was indicative of both overall survival (OS) and disease-free survival (DFS), with a shorter survival for the Fibrosis patients (Table 3). In contrast, while the D-I-M-P signature was indicative of overall survival, it had no prognostic value for disease-free survival in the univariate analysis (Table 3). In the multivariate analysis, none of the transcriptomic stratifications (either into two or four subgroups) were associated with overall survival, independent of stage and debulking (Table 3). Still, the Fibrosis – non-Fibrosis signature was the only one to be independent of stage and debulking and to provide additive prognostic value for disease-free survival (Table 3).

**TABLE 3:** Stratification of high-grade serous ovarian cancers into two subgroups provides a prognostic value, independent of stage and debulking.

	**OS univariate analysis**					**OS multivariate analysis**				

	**HR**	**CI 95% inf**	**CI 95% sup**	***p*-value**		**HR**	**CI 95% inf**	**CI 95% sup**	***p*-value**	
**Signature**										
Non-Fibrosis	Ref					Ref				
Fibrosis	1.43	1.13	1.82	**0.003**	**	1.22	0.95	1.57	0.12	
**Stage**										
II	Ref					Ref				
III	2.49	1.17	5.29	**0.02**	*	2.39	0.97	5.87	0.06	
IV	3.28	1.49	7.25	**0.003**	**	2.82	1.11	7.18	**0.03**	*
**Debulking**										
Full	Ref					Ref				
Partial	2.01	1.37	2.94	**0.0004**	***	1.90	1.27	2.82	**0.002**	**
**Age**										
<59 years	Ref									
>59 years	1.2	0.96	1.55	0.11						
**Signature**										
D	1.48	1.03	2.14	**0.04**	*	1.23	0.83	1.80	0.30	
I	Ref					Ref				
M	1.67	1.13	2.47	**0.01**	**	1.12	0.74	1.69	0.59	
P	1.40	0.96	2.03	0.08		1.17	0.79	1.72	0.43	
**Stage**										
II	Ref					Ref				
III	2.49	1.17	5.29	**0.02**	*	2.44	0.99	6.00	**0.05**	
IV	3.28	1.49	7.25	**0.003**	**	2.72	1.06	6.96	**0.04**	*
**Debulking**										
Full	Ref					Res				
Partial	2.01	1.37	2.94	**0.0004**	***	1.92	1.29	2.86	**0.001**	**

	**DFS univariate analysis**					**DFS multivariate analysis**				

	**HR**	**CI 95% inf**	**CI 95% sup**	***p*-value**		**HR**	**CI 95% inf**	**CI 95% sup**	***p*-value**	

**Signature**										
Non-Fibrosis	Ref					Ref				
Fibrosis	1.37	1.08	1.73	**0.01**	*	1.28	0.99	1.65	**0.05**	
**Stage**										
II	Ref					Ref				
III	1.93	1.12	3.31	**0.02**	*	1.67	0.89	3.12	0.11	
IV	2.48	1.35	4.54	**0.003**	**	2.03	1.02	4.04	**0.05**	*
**Debulking**										
Full	Ref					Ref				
Partial	1.69	1.23	2.32	**0.001**	**	1.54	1.11	2.13	**0.01**	*
**Age**										
<59 years	Ref									
>59 years	0.95	0.75	1.2	0.7						
**Signature**										
D	1.32	0.94	1.86	0.11		1.19	0.84	1.72	0.35	
I	Ref					Ref				
M	1.41	0.97	2.04	0.07		1.14	0.76	1.69	0.53	
P	1.23	0.87	1.74	0.24		1.12	0.78	1.63	0.54	
**Stage**										
II	Ref					Ref				
III	1.93	1.12	3.31	**0.02**	*	1.73	0.93	3.24	0.09	
IV	2.48	1.35	4.54	**0.003**	**	2.11	1.06	4.21	**0.03**	*
**Debulking**										
Full	Ref					Ref				
Partial	1.69	1.23	2.32	**0.001**	**	1.54	1.11	2.14	**0.01**	**

*Cox proportional hazards regression was performed on Fibrosis/non-Fibrosis or D-I-M-P subgroups and evaluated for overall survival (OS, Top) and disease-free survival (DFS, Bottom). These analyses were either: adjusted for stage and debulking status (multivariate, Right) or unadjusted (univariate, Left). Age at diagnosis was not taken into account for multivariate analysis as it was not significant at univariate level. HR, hazard ratio; CI 95% inf, lower limit of the 95% confidence interval; CI 95% sup, upper limit of the 95% confidence interval. Significant p-values are indicated in bold.* *p ≤ 0.05, **p ≤ 0.01, ***p ≤ 0.001.

**FIGURE 2:**
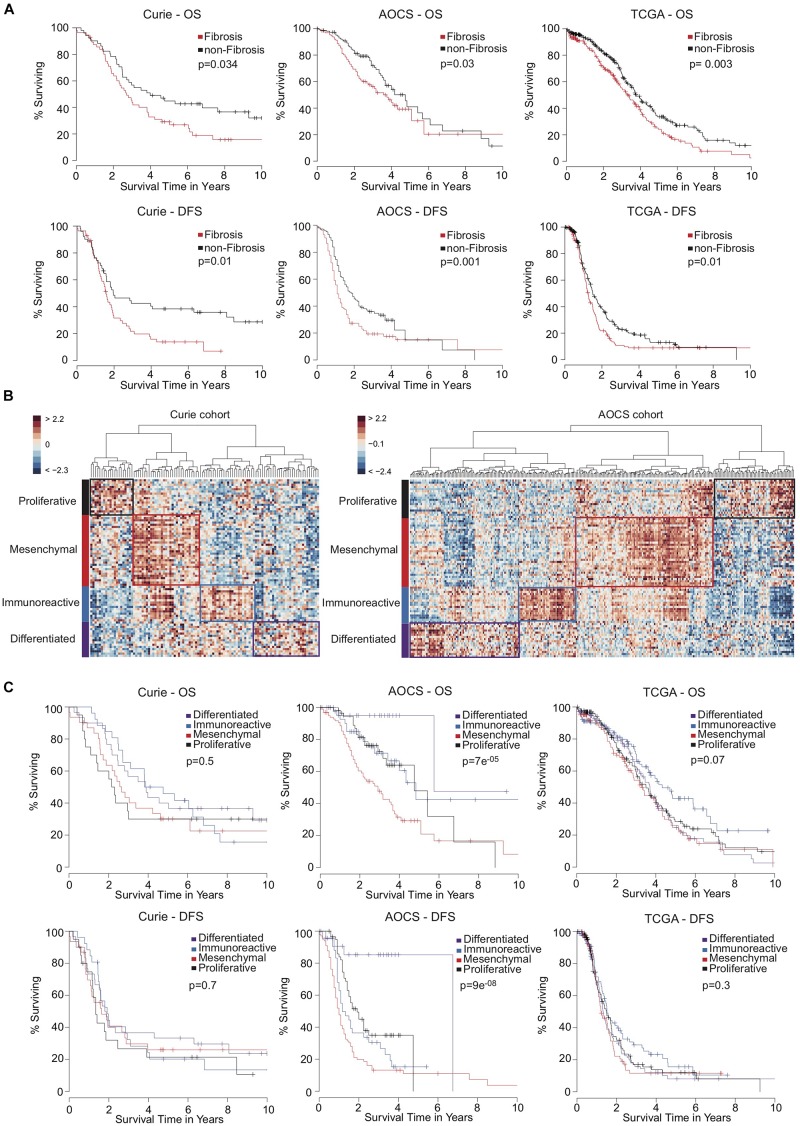
High-grade serous ovarian cancers stratified into two transcriptomic subgroups exhibit a reliable prognostic value of patient survival. **(A)** Kaplan-Meier curves showing 10-year overall survival (OS, Top) and disease-free survival (DFS, Bottom) of patients with Fibrosis (red) or non-Fibrosis (black) ovarian cancers. Patients from the Curie cohort (56 Fibrosis and 51 non-Fibrosis), the AOCS cohort (135 Fibrosis and 150 non-Fibrosis) and the TCGA cohort (220 Fibrosis and 326 non-Fibrosis) were analyzed, as indicated. *P*-values were calculated using the Log-rank test. **(B)** Heatmap and hierarchical clustering applied on the Curie (Left) and AOCS (Right) cohorts. Rows represent genes and columns represent patients. Clustering is based on the 100 genes of the D-I-M-P signature (Verhaak et al., 2013) using Euclidean distance and Ward’s agglomeration method. The color saturation shows the magnitude of the deviation from the mean for each gene, with red and blue indicating expression values above or below the mean, respectively. **(C)** Kaplan-Meier curves showing 10-year overall survival (OS, Top) and disease-free survival (DFS, Bottom) of ovarian cancer patients according to D-I-M-P classification. Patients from the Curie (*N* = 30 Differentiated, *N* = 26 Immunoreactive, *N* = 31 Mesenchymal, and *N* = 20 Proliferative), the AOCS (*N* = 25 Differentiated, *N* = 42 Immunoreactive, *N* = 102 Mesenchymal, and *N* = 60 Proliferative) and the TCGA (*N* = 148 Differentiated, *N* = 129 Immunoreactive, *N* = 118 Mesenchymal, and *N* = 138 Proliferative) cohorts were analyzed. *P*-values were calculated using the Log-rank test.

In the Kaplan-Meier survival analyses, Fibrosis patients exhibited significantly shorter overall survival (Figure 2A, Top) and disease-free survival (Figure 2A, Bottom) than non-Fibrosis patients in the three independent cohorts analyzed (Curie, AOCS, and TCGA). Classification using the D-I-M-P signature was initially only performed in the TCGA cohort (Verhaak et al., 2013). Therefore, we used unsupervised clustering to identify the four D-I-M-P subgroups in the Curie and AOCS cohorts (Figure 2B). The classification into those four subgroups was prognostic factor for overall survival and disease-free survival in the AOCS cohort, but not in the Curie and TCGA cohorts (Figure 2C). This shows that the D-I-M-P signature does not provide a systematic prognostic value for survival of ovarian cancer patients, but the division into two molecular subgroups, Fibrosis and non-Fibrosis, is discriminant and reliable. Because the Fibrosis/non-Fibrosis signature was defined by genes correlated- or anti-correlated with miR-200 expression (Mateescu et al., 2011; Batista et al., 2013, 2016), we also evaluated their prognostic value. No microRNA, separately or in combination, was sufficient as a prognostic marker for patient survival (Supplementary Figure S3), indicating that expression of the miR-200 family is not an applicable surrogate marker of patient outcome. In conclusion, stratification of ovarian cancer patients into two subgroups using the Fibrosis/non-Fibrosis signature provides a reliable prognostic value for patient survival, but using the D-I-M-P signature or miR-200 family member expression levels do not.

### LST Genomic Signature Identifies Ovarian Cancer With HRD

High-grade serous ovarian cancers were analyzed according to the LST genomic signature allowing us to stratify patients into two subgroups: high LST (LST^Hi^) for HRD tumors (303 tumors, 56%) or low LST (LST^Lo^) for non-HRD tumors (238 tumors, 44%). As expected, LST^Hi^ ovarian cancers were associated with BRCA1/2 mutations, BRCA1 or RAD51C promotor methylation and showed increased sensitivity to platinum-based chemotherapy (Table 4). In contrast, the LST signature was not significantly associated with grade, debulking status or primary therapy outcome (Table 4). Univariate analyses, using the Cox regression model, showed that LST signature was indicative of better survival for LST^Hi^ patients (*p* = 5.4 × 10^–10^ for overall survival and *p* = 1.7 × 10^–5^ for disease-free survival). BRCA1/2 mutations were also associated with better patient outcome (*p* = 1.8 × 10^–4^ for overall survival and *p* = 0.01 for disease-free survival), but methylation of BRCA1 and RAD51C promoter regions were not. In multivariate Cox analyses adjusted for BRCA1/2 mutations, LST^Lo^ patients remained significantly associated with shorter disease-free survival (HR = 1.6, CI95% [1.2–2.1], *p* = 3.9 × 10^–4^, with HR, Hazard Ratio and CI, Confidence Interval) and overall survival (HR = 1.95, CI 95% [1.5–2.5], *p* = 6.7 × 10^–7^), whereas the presence of a BRCA mutation was not associated with disease-free survival (*p* = 0.34) and much less associated with overall survival (*p* = 0.04). This shows that using the LST signature is more efficient for predicting survival of ovarian cancer patients than testing the presence of BRCA1/2 mutations.

**TABLE 4:** Association between genomic signature and clinical parameters.

		**LST^Lo^**	**LST^Hi^**	***p*-value**
Grade				*p* = 0.89
	G2	27 (12.5%)	30 (11.8%)	
	G3	189 (87.5%)	224 (88.2%)	
Stage				*p* = 0.29
	II	8 (3.7%)	16 (6.2%)	
	III–IV	210 (96.3%)	243 (93.8%)	
Debulking				*p* = 0.55
	Full	39 (19.8%)	51 (22.4%)	
	Partial	158 (80.2%)	177 (77.6%)	
Platinum resistance				***p* = 0.0001**
	Sensitive	71 (56.3%)	124 (78.5%)	
	Resistant	55 (43.7%)	34 (21.5%)	
Primary therapy outcome				*p* = 0.07
	Complete response	111 (65.3%)	164 (73.9%)	
	Partial response	59 (34.7%)	58 (26.1%)	
BRCA1/2 mutation				***p* = 2.0 × 10^–15^**
	No	229 (96.2%)	217 (71.6%)	
	Yes	9 (3.8%)	86 (28.4%)	
BRCA1 methylation				***p* = 1.9 × 10^–19^**
	No	238 (100%)	235 (77.6%)	
	Yes	0 (0%)	68 (22.4%)	
RAD51C methylation				***p* = 0.0008**
	No	238 (100%)	290 (95.7%)	
	Yes	0 (0%)	13 (4.3%)	
Transcriptomic signature				*p* = 0.29
	Non-Fibrosis	147 (62.3%)	171 (57.4%)	
	Fibrosis	89 (37.7%)	127 (42.6%)	
Ploidy				***p* = 2.7 × 10^–15^**
	2	41 (17.2%)	150 (49.5%)	
	≥ 4	197(82.8%)	153 (50.5%)	

**Contingency table showing associations between the LST^Lo^/LST^Hi^ subgroups and clinical parameters. Debulking status and response to primary therapy were defined as in Table 2. These analyses were performed on the TCGA cohort. P-values were calculated using Fisher’s exact test and significant p-values are indicated in bold.**

### Genomic and Transcriptomic Signatures Provide Additive Prognostic Values for Ovarian Cancer Patient Survival

**FIGURE 3:**
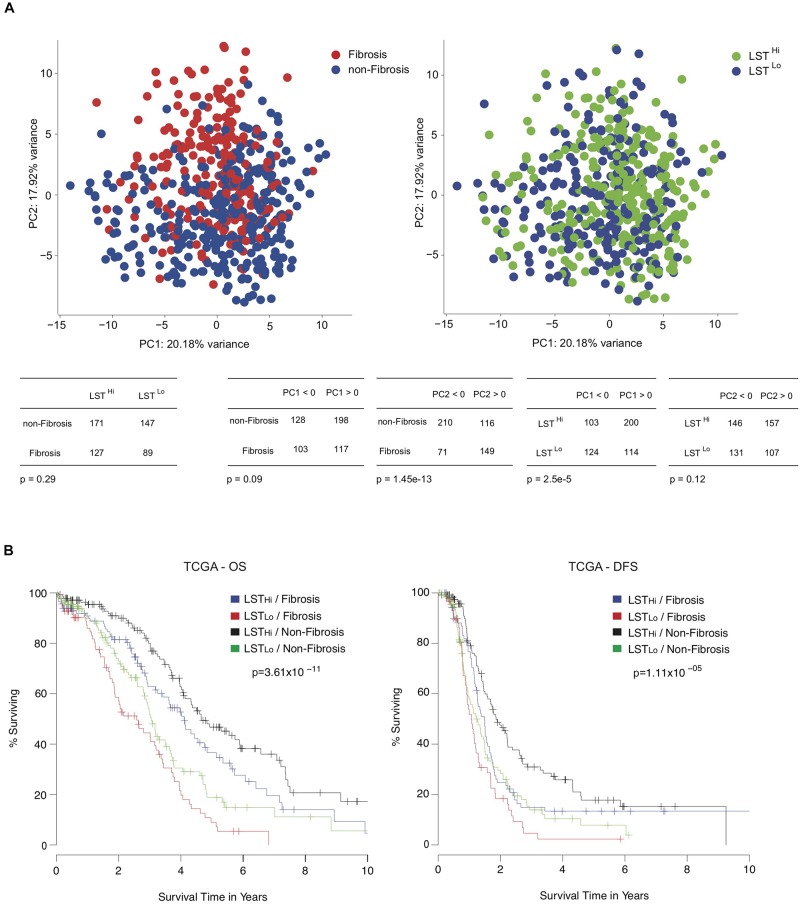
Combining genomic and transcriptomic signatures provide additive prognostic values for high-grade serous ovarian cancer patients. (**A**, Up) Principal component analyses (PCA) applied on transcriptomic data from the TCGA cohort using Verhaak’s signature. On the left panel, the color code shows the non-Fibrosis (blue, *N* = 326) and Fibrosis (red, *N* = 220) subgroups, using Mateescu’s signature (Mateescu et al., 2011). The right panel shows the same PCA representation but subgroups are highlighted using the LST genomic signature (Popova et al., 2012). The color code represents LST^Lo^ (blue, *N* = 238) and LST^Hi^ (green, *N* = 303) ovarian cancers. (Down) Contingency tables showing the repartition of patients regarding Mateescu or LST classification and the repartition against the two first principal components. **(B)** Kaplan-Meier curves showing 10-year overall survival (OS, Left) and disease-free survival (DFS, Right), after stratification into four groups: LST^Lo^/Fibrosis (red, *N* = 89), LST^Lo^/non-Fibrosis (green, *N* = 147), LST^Hi^/Fibrosis (blue, *N* = 127), and LST^Hi^/non-Fibrosis (black, *N* = 171). *P*-values are calculated using the Log-rank test.

As shown above, the LST signature was significantly associated with HRD and platinum-sensitivity. In contrast, the Fibrosis/non-Fibrosis signature was linked to stage and clinical response to treatment, suggesting these signatures could be complementary. Performing Principal Component Analyses (PCA) on the TCGA transcriptomic data (Verhaak et al., 2013), we observed that the Fibrosis/non-Fibrosis signature did not overlap with the LST signature (Figure 3A, Top) and this lack of association was also statistically confirmed (*p* = 0.29, Table 2). Interestingly, the two signatures were not associated with the same principal components (PC): Fibrosis/non-Fibrosis signature was found associated with PC2 (*p* < 2.2 × 10^–16^) while the LST signature was found associated with PC1 (*p* = 2.1 × 10^–6^) (Figure 3A, Bottom). We then investigated if, together, they could provide additive value regarding prognosis. Interestingly, the genomic (LST) and transcriptomic (Fibrosis-/non-Fibrosis) signatures were complementary and defined four distinct patient subgroups with significantly different survival (Figure 3B). In other words, Fibrosis and non-Fibrosis patients could be subdivided into LST^Hi^ and LST^Lo^ subgroups. As expected, the Fibrosis subtype was associated with poor prognosis, in particular when combined to LST^Lo^, the non-HRD status (Figure 3B). Reciprocally, the non-Fibrosis patients were characterized by a better outcome, especially when associated with the LST^Hi^ subgroup (Figure 3B). Pairwise comparison showed that each subgroup was significantly different from each other, in term of overall survival and disease-free survival (apart from the LST^Lo^/non-Fibrosis subgroup in the disease-free survival analyses) (Table 5). These data show that combining genomic and transcriptomic signatures improved stratification of high-grade serous ovarian cancers and provided a significant additive prognostic value. These two signatures (genomic and transcriptomic) were independent for predicting disease-free survival (LST: HR = 1.7, CI 95% [1.3–2.2], *p* = 10^–5^; Fibrosis/non-Fibrosis: HR = 1.4, CI 95% [1.1–1.8], *p* = 6 × 10^–3^ by multivariate Cox regression analysis) and overall survival (LST: HR = 2.2, CI 95% [1.7–2.8]; *p* = 4 × 10^–10^; Fibrosis/non-Fibrosis: HR = 1.5, CI 95% [1.2–2], *p* = 1 × 10^–3^). In contrast to the Fibrosis/non-Fibrosis signature, the D-I-M-P and LST signatures were significantly associated (*p* = 0.02, Table 2). The multivariate Cox analysis adjusted for the D-I-M-P and LST signatures showed that the two signatures were independent for predicting overall survival (LST: HR = 2.16, CI 95% [1.7–2.8], *p* = 2.3 × 10^–9^; DIMP: HR = 1.57, CI 95% [1.1–2.3], *p* = 0.02). In contrast, only the LST signature was significantly associated with the disease-free survival (LST: HR = 1.71, CI 95% [1.3–2.2], *p* = 2.3 × 10^–5^), while D-I-M-P was not. In conclusion, these results demonstrate that combining genomic and transcriptomic information is the most reliable method for stratifying high-grade serous ovarian cancer patients.

**TABLE 5:** Pairwise comparison of transcriptomic and genomic signatures for overall and disease-free survival.

	**LST^Hi^/Fibrosis**	**LST^Lo^/Fibrosis**	**LST^Hi^/Non-Fibrosis**	**LST^Lo^/Non-Fibrosis**
**Overall Survival**				
LST^Hi^/Fibrosis	–			
LST^Lo^/Fibrosis	1.60 × 10^–0.5^	–		
LST^Hi^/Non-Fibrosis	0.03	1.00 × 10^–11^	–	
LST^Lo^/Non-Fibrosis	0.04	0.03	1.10 × 10^–05^	–
**Disease Free Survival**				
LST^Hi^/Fibrosis	–			
LST^Lo^/Fibrosis	0.03	–		
LST^Hi^/Non-Fibrosis	0.03	2.60 × 10^–0.6^	–	
LST^Lo^/Non-Fibrosis	0.31	0.14	7.1 × 10^–0.4^	–

**Differences in overall survival (Top) and disease-free survival (Bottom) between the four groups: LST^Hi^/Fibrosis, LST^Lo^/Fibrosis, LST^Hi^/Non-Fibrosis and LST^Lo^/Non-Fibrosis. P-values are calculated using the Log-rank test and corrected for multiple testing using the Benjamini-Hochberg procedure.**

## Discussion

Stratification of high-grade serous ovarian cancer patients remains unclear. Previously, several ovarian cancer molecular subgroups were identified according to transcriptomic signatures (Tothill et al., 2008; Cancer Genome and Atlas Research, 2011; Mateescu et al., 2011; Sabatier et al., 2011; Bentink et al., 2012; Verhaak et al., 2013; Konecny et al., 2014) or genomic (Fong et al., 2009, 2010; Audeh et al., 2010; Goundiam et al., 2015). In this study, we define the optimal number of ovarian cancer molecular subgroups with reproducible prognostic value. The study of several independent cohorts showed that classifying ovarian tumors into four molecular subgroups, based on D-I-M-P signatures, does not reproducibly inform on patient survival. In contrast, the subdivision of patients into two molecular subgroups (Fibrosis/non-Fibrosis) provided reliable prediction of patient survival. We also identified a novel complementarity between transcriptomic and genomic data. Indeed, transcriptomic profiling and HRD status characterize specific biological processes and could accurately reflect the different key components in ovarian tumors. Furthermore, combining both genomic and transcriptomic data identified four ovarian cancer patient subgroups with distinct prognostic values and is, therefore, currently the most appropriate method for stratifying high-grade serous ovarian cancer patients.

Although several transcriptomic signatures in ovarian cancers have been proposed (Tothill et al., 2008; Cancer Genome and Atlas Research, 2011; Mateescu et al., 2011; Sabatier et al., 2011; Bentink et al., 2012; Verhaak et al., 2013; Konecny et al., 2014), there is no clear consensus for choosing a specific one. This is mainly due to the lack of overlap in the gene sets of these transcriptomic signatures (Tothill et al., 2008; Cancer Genome and Atlas Research, 2011; Mateescu et al., 2011; Sabatier et al., 2011; Bentink et al., 2012; Verhaak et al., 2013; Konecny et al., 2014). The lack of overlap could be explained, at least in part, by the heterogeneity in the techniques and platforms used for detecting gene expression, and by the diversity of unsupervised algorithms applied to molecular classifications. There is a clearer consensus of molecular classifications in breast cancer (Perou et al., 2000; Sorlie et al., 2001; Coates et al., 2015) that could be due to the presence of confirmed biomarkers (for example, hormonal receptors, and HER2 expression). The lack of consistency found in ovarian cancer classifications highlights the importance of using appropriate methods for stratifying high-grade serous ovarian cancer patients. Here, we demonstrate that among the four transcriptomic signatures analyzed (Tothill et al., 2008; Cancer Genome and Atlas Research, 2011; Mateescu et al., 2011; Bentink et al., 2012; Verhaak et al., 2013), patient stratification into two subgroups, defined as the Fibrosis/non-Fibrosis signature (Mateescu et al., 2011), exhibits the most reliable prognostic value for patient survival compared to the others. We did observe a significant overlap in patient classification by applying the different transcriptomic signatures analyzed, but we also detected some differences between classifications. Indeed, Mesenchymal patients defined by the D-I-M-P signature (Verhaak et al., 2013) were all identified as Fibrosis using Mateescu’s signature (Mateescu et al., 2011). In contrast, some patients defined as non-Mesenchymal by the D-I-M-P signature were defined as Fibrosis using Mateescu’s signature, and they also exhibited poor survival. Based on the survival-data analyses, these observations suggest that some non-Mesenchymal patients should be considered Mesenchymal, as determined by the Fibrosis signature. This could also be explained by the non-exclusive attribution to a subtype using the D-I-M-P signature (40% of the tumor samples could be assigned to two distinct subtypes in Konecny’s study) (Konecny et al., 2014) and/or by the spatial heterogeneity of signatures caused by the different geographic areas of sampling. Importantly, classifications tested in these studies (Tothill et al., 2008; Cancer Genome and Atlas Research, 2011; Bentink et al., 2012; Verhaak et al., 2013) were defined using a similar methodology (non-supervised analysis), but the Fibrosis/non-Fibrosis signature was identified through mechanistic studies based on miR-200-dependent profiling (Mateescu et al., 2011; Batista et al., 2016). This may explain the heterogeneity seen between our signature and others. In addition, our observations indicated that expression of miR-200 family members, either separately or combined, was not sufficient to predict patient survival. There has been a long-lasting controversy about the prognostic value of miR-200 with a number of studies displaying divergent results (Batista et al., 2013; Muralidhar and Barbolina, 2015). Recently, a meta-analysis including 7 articles with available data (553 patients) was conducted (Shi and Zhang, 2016). It is important to note that the populations included in those studies were quite small (from 55 to 100 patients) compared to the TCGA cohort studied here (557 patients). In that meta-analysis, higher expression of the miR-200 family was significantly associated with improved survival, predominantly due to the impact of miR-200c. This association was stronger in the Asian population. The discrepancies between this meta-analysis and our findings may be due to several reasons: inclusion of less Asian patients in the TCGA cohort, multiple small studies using different microarray protocols and significant heterogeneity across studies in the meta-analysis. This indicates that the prognostic value of using expression of the miR-200 family lacks reliability. Nonetheless, circulating miR-200s could still be good indicators for early detection of ovarian cancers or dynamic markers to follow-up during chemotherapy, as suggested in previous studies (Taylor and Gercel-Taylor, 2008; Kan et al., 2012; Sarojini et al., 2012; Kapetanakis et al., 2015; Pendlebury et al., 2017).

In addition to transcriptomic data, we have here provided new insight into genomic signatures of ovarian cancers. LST, defined as chromosomal breaks between adjacent regions of at least 10 Mb, constitute a robust indicator of HRD status (Popova et al., 2012; Goundiam et al., 2015). This classification was initially defined in breast cancers (Popova et al., 2012). Triple-negative breast carcinomas and high-grade serous ovarian cancers have some genomic instability patterns in common, providing a strong rationale for applying this LST signature on ovarian cancers. We and others have shown the impact of HRD on favorable response to platinum salts and overall survival (Fong et al., 2009, 2010; Audeh et al., 2010; Popova et al., 2012; Goundiam et al., 2015; Manie et al., 2016). Here, we confirm the clear prognostic value of the LST signature in high-grade serous ovarian cancers with better survival demonstrated for LST^Hi^ patients. Moreover, the interest for this classification will probably increase with the inclusion of PARP-inhibitors in routine clinical practice. Currently, the same therapeutic strategy, a combination of platinum and taxane-based chemotherapy, is used for all patients suffering from high-grade ovarian cancers. In the last decade, anti-angiogenic therapies and PARP-inhibitors were approved for treatment of high-grade ovarian cancers, with a significant but limited impact on survival. This benefit on survival may be hidden by the molecular heterogeneity in tumors that drives either beneficial or deleterious response to treatments. Recent findings suggest that transcriptomic signatures could help in the identification of patients who will benefit from anti-angiogenic therapies (Gourley et al., 2014; Kommoss et al., 2017). In that context, we propose stratification of ovarian cancer patients that could help identify different sensitivity to treatment. The duality of our signature considering both the genomic HRD profile (LST signature) and the transcriptomic microenvironment features (Fibrosis/non-Fibrosis signature) provides compelling data for new therapies targeting the microenvironment (Thibault et al., 2014). There is a tendency to limit reimbursement of expansive new therapies if there is no biomarker predicting treatment response. We provide a reliable method to identify and subgroup high-grade serous ovarian cancer patients by combining genomic and transcriptomic information. Thus, our proposition of stratification could be used as a biomarker for some therapies that may help clinicians define the most appropriate therapeutic strategy.

## Data Availability Statement

Publicly available datasets were analyzed in this study. This data can be found here: GSE26193 and GSE9899, Clinical characteristics of the 557 patients included in the TCGA cohort, as well as transcriptomic data generated using Affymetrix Human Genome U133A arrays, have been previously described in Cancer Genome and Atlas Research (2011) and can be downloaded from the NIH Genomic Data Commons (GDC) data portal (https://gdc-portal.nci.nih.gov).

## Ethics Statement

The studies involving human participants were reviewed and approved by The Institutional Review Board and Ethics committee of the Institut Curie Hospital Group approved all analyses realized in this study. Written informed consent for participation was not required for this study in accordance with the national legislation and the institutional requirements.

## Author Contributions

FM-G and YK participated in the conception and design of the study. YK performed bioinformatic and statistical analyses of the data, with participation from CB. TP and M-HS defined the LST status of high-grade serous ovarian cancers. RR provided human samples from the Curie cohort. FM-G supervised the entire project and wrote the manuscript with YK and CB.

## Conflict of Interest

TP and M-HS are named inventors of a patent for the genomic signature of BRCAness. Current exploitation of the patent is on-going by Myriad Genetics. The remaining authors declare that the research was conducted in the absence of any commercial or financial relationships that could be construed as a potential conflict of interest.

## Footnotes

1 https://www.ncbi.nlm.nih.gov/geo/query/acc.cgi?acc=GSE26193

2 https://portal.gdc.cancer.gov

3 https://david.ncifcrf.gov

4 http://revigo.irb.hr

5 https://cran.r-project.org
