# Supplementary material for: Clinical Interest of Combining Transcriptomic and Genomic Signatures in High-Grade Serous Ovarian Cancer
Source: Front Genet. 2020 Mar 17;11:219. doi: 10.3389/fgene.2020.00219 (PMC7089941; doi:10.3389/fgene.2020.00219)
Supplement: Supplementary file 1 [file Data_Sheet_1.PDF]

*Supplementary Material for*

# Clinical interest of combining transcriptomic and genomic signatures in high-grade serous ovarian cancer

**Yann Kieffer[1,2#], Claire Bonneau[1,2#], Tatiana Popova[2,3], Roman Rouzier[4], Marc-Henri Stern[2,3] and Fatima Mechta-Grigoriou[1,2*]**

[#] These authors contributed equally to this work

*** Correspondence:**
Corresponding Author: Dr. Fatima Mechta-Grigoriou, ORCID Number: 0000-0002-3751-6989, Phone: +33 (0)1 56 24 66 53, Fax: +33 (0)1 56 24 66 50, Email address: fatima.mechta-grigoriou@curie.fr

*Corresponding author: Dr. Fatima Mechta-Grigoriou, ORCID Number: 0000-0002-3751-6989, Phone: +33 (0)1 56 24 66 53, Fax: +33 (0)1 56 24 66 50, Email address: fatima.mechta-grigoriou@curie.fr

## SUPPLEMENTARY INFORMATION LEGENDS

**Supplementary Figure 1: The Fibrosis subgroup of high-grade serous ovarian cancers exhibits conserved functional pathways across studies**

**A.** Pathway enrichment analysis showing top significant and non-redundant Gene Ontology (GO) terms in the C1, Fibrosis, Angiogenic and Mesenchymal signatures. **B.** Venn diagram showing overlapping genes between the C1, Fibrosis, Angiogenic and Mesenchymal signatures. **C.** Pathway enrichment analysis showing top significant and non-redundant GO terms in non-fibrosis signatures, such as C2-C6, stress, M2-M4 and D-I-P. Abbreviations are as follows: reg: regulation; loc: localization; pos: positive; neg: negative. **D.** Venn diagram showing overlapping genes between non-Fibrosis Fibrosis signatures.

**Supplementary Figure 2: High-grade serous ovarian cancer patients classified as Fibrosis/non-Mesenchymal and Fibrosis/Mesenchymal show a similar poor prognosis**

**A.** Kaplan-Meier curves showing 10-year overall survival (OS, **Left**) and disease-free survival (DFS, **Right**) of patients with ovarian tumors classified as Fibrosis/Mesenchymal (red, N=108), Fibrosis/non-Mesenchymal (purple, N=108) or non-Fibrosis/non-Mesenchymal (black, N=307), according to the Mateescu and Verhaak classifications. These analyses were performed on the TCGA cohort, as indicated. P-values were calculated using the Log-rank test.

**Supplementary Figure 3: miR-200 family members are not prognostic for ovarian cancer patient survival**

**A.** Kaplan-Meier curves showing 10-year overall survival of patients suffering from high-grade serous ovarian cancer with either high expression (red, N=242) or low expression (black, N=241) of miR-200 family members. The threshold between low and high microRNA expression subclasses is based on the median expression of each microRNA individually, as indicated, or on the median of the expression average of all miR-200 family members (**Bottom right**). These analyses were performed on the TCGA cohort and p-values were calculated using the Log-rank test.

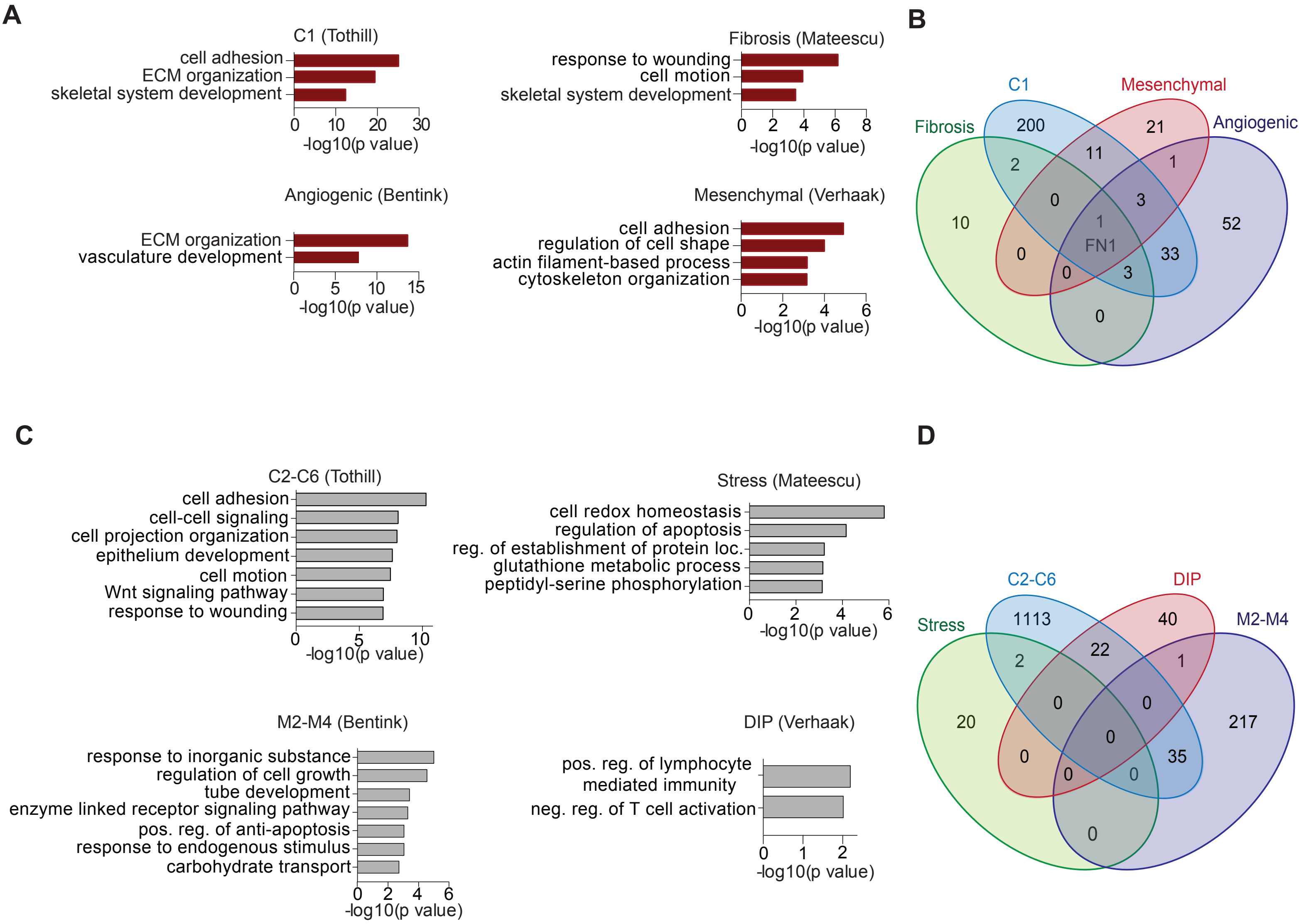
A
C1 (Tothill)
cell adhesion
ECM organization
skeletal system development
0
10
20
30
-log10(p value)
Fibrosis (Mateescu)
response to wounding
cell motion
skeletal system development
0
2
4
6
8
-log10(p value)
Angiogenic (Bentink)
ECM organization
vasculature development
0
5
10
15
-log10(p value)
Mesenchymal (Verhaak)
cell adhesion
regulation of cell shape
actin filament-based process
cytoskeleton organization
0
2
4
6
-log10(p value)
B
C1
Mesenchymal
Fibrosis
Angiogenic
200
21
11
2
1
0
3
10
1
FN1
52
0
33
0
3
0
C
C2-C6 (Tothill)
cell adhesion
cell-cell signaling
cell projection organization
epithelium development
cell motion
Wnt signaling pathway
response to wounding
0
5
10
-log10(p value)
Stress (Mateescu)
cell redox homeostasis
regulation of apoptosis
reg. of establishment of protein loc.
glutathione metabolic process
peptidyl-serine phosphorylation
0
2
4
6
-log10(p value)
M2-M4 (Bentink)
response to inorganic substance
regulation of cell growth
tube development
enzyme linked receptor signaling pathway
pos. reg. of anti-apoptosis
response to endogenous stimulus
carbohydrate transport
0
2
4
6
-log10(p value)
DIP (Verhaak)
pos. reg. of lymphocyte
mediated immunity
neg. reg. of T cell activation
0
1
2
-log10(p value)
D
C2-C6
DIP
Stress
M2-M4
1113
40
22
2
1
0
0
20
0
217
0
35
0
0
0

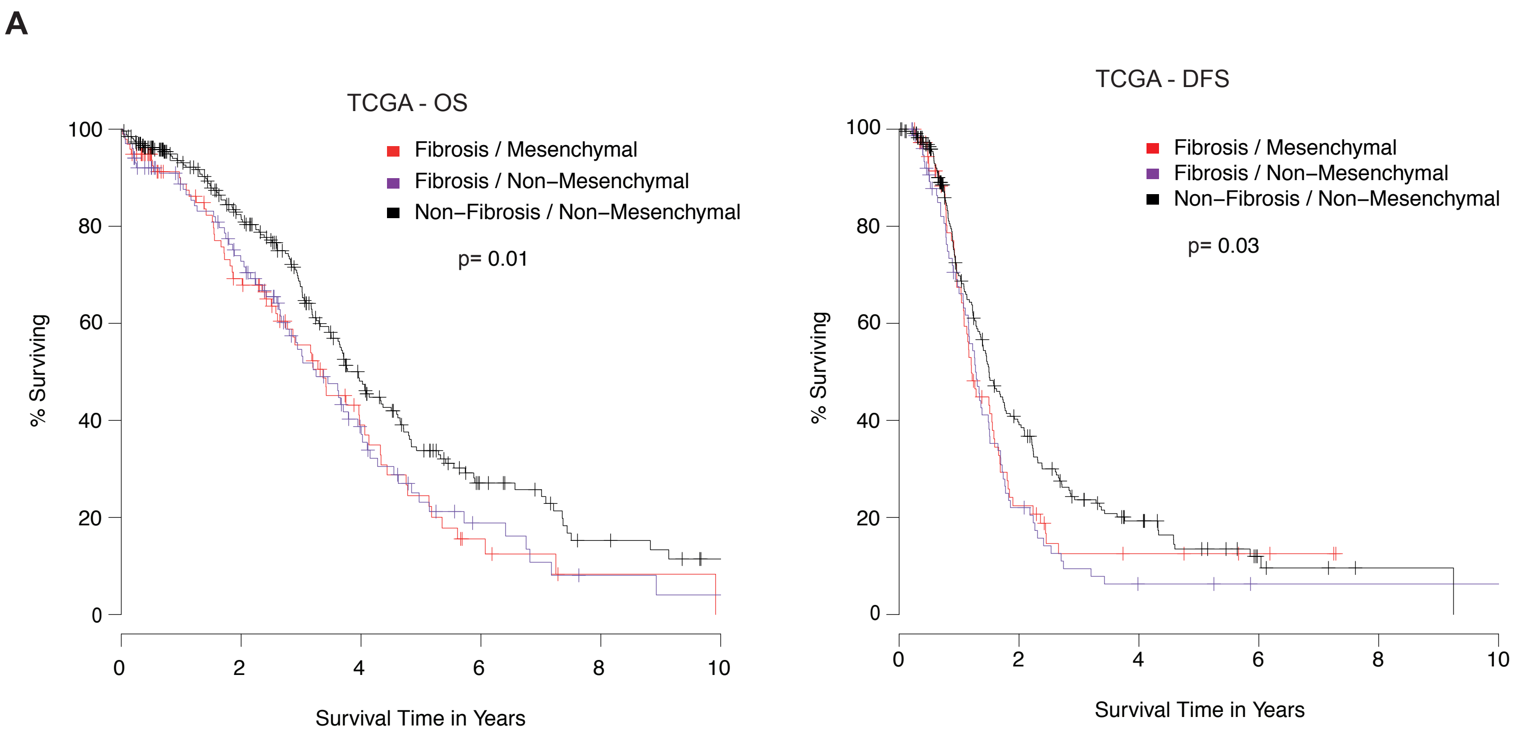
A
TCGA - OS
Fibrosis / Mesenchymal
Fibrosis / Non–Mesenchymal
Non–Fibrosis / Non–Mesenchymal
p= 0.01
% Surviving
Survival Time in Years
TCGA - DFS
Fibrosis / Mesenchymal
Fibrosis / Non–Mesenchymal
Non–Fibrosis / Non–Mesenchymal
p= 0.03
% Surviving
Survival Time in Years

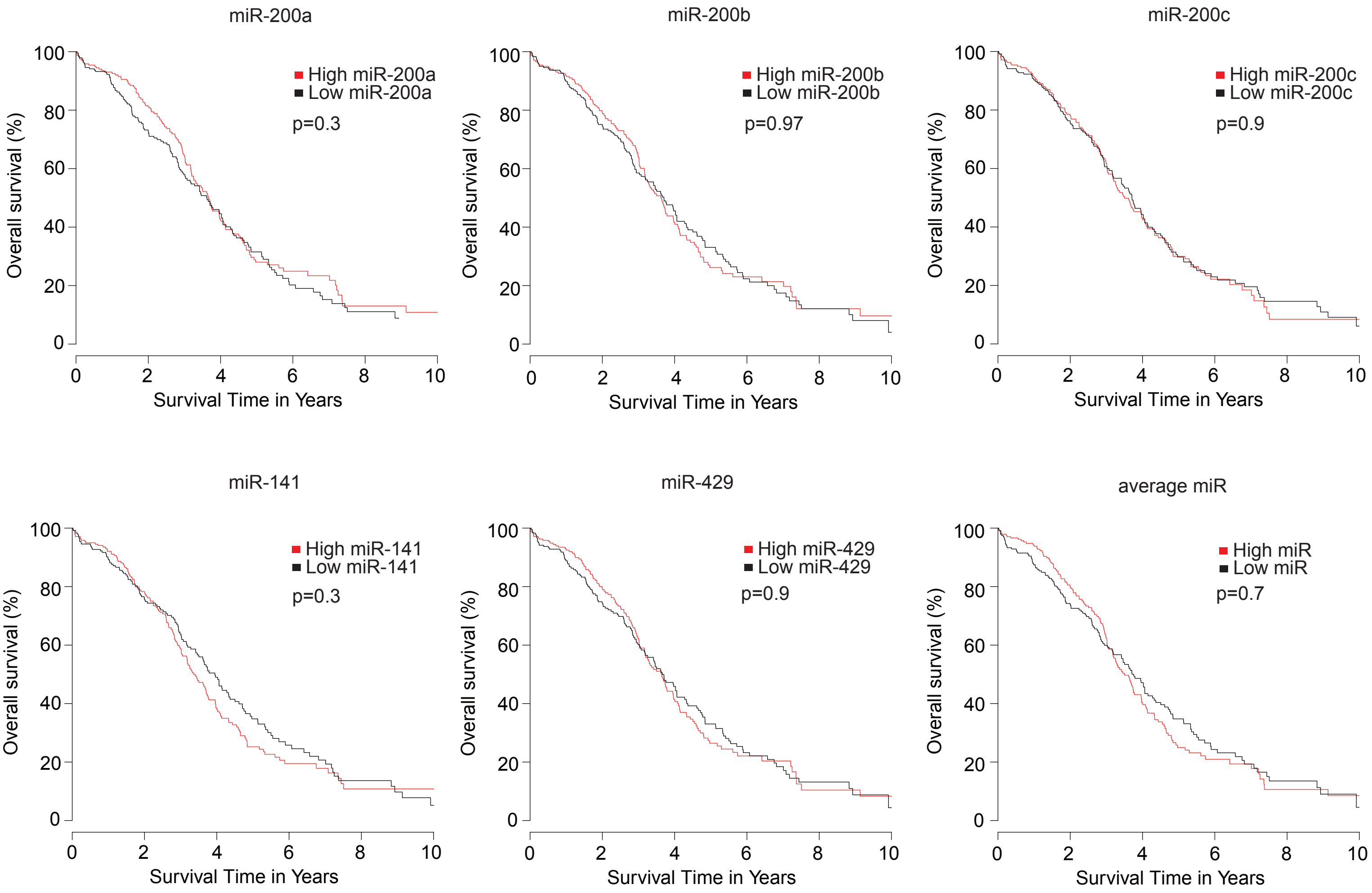
miR-200a
High miR-200a
Low miR-200a
p=0.3
Overall survival (%)
Survival Time in Years
miR-200b
High miR-200b
Low miR-200b
p=0.97
miR-200c
High miR-200c
Low miR-200c
p=0.9
miR-141
High miR-141
Low miR-141
p=0.3
miR-429
High miR-429
Low miR-429
p=0.9
average miR
High miR
Low miR
p=0.7
